# Evidence-Based Prescribing of Statins: A Developing World Perspective

**DOI:** 10.1371/journal.pmed.0030050

**Published:** 2006-02-21

**Authors:** H. T Ong

## Abstract

Although statins reduce the incidence of cardiovascular disease, they are expensive. How should trial evidence on statins be applied to a resource-poor setting?

Over the last decade, landmark clinical trials (summarized in Tables 1 and 2) have shown that statin therapy reduces coronary events in patients with or without prior cardiovascular disease [ 1– 12]. More recent studies, summarized in Table 3, show that the larger the statin dosage, the greater the reduction in cardiovascular clinical events [ 13– 17]. A meta-analysis involving 90,056 patients in 14 randomized trials emphasizes that the bene. t of statin treatment is not limited to a reduction in coronary disease; treatment also reduces the incidence of strokes, coronary revascularization, and coronary and total mortality [ 18].

**Table 1:**
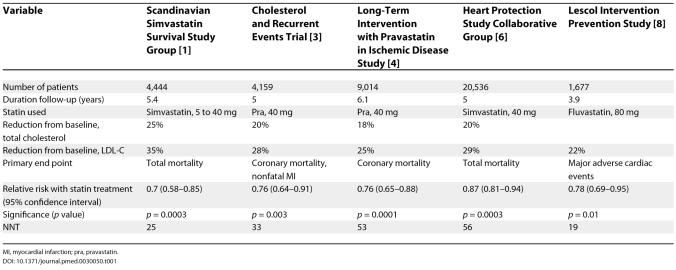
Placebo-Controlled Secondary Prevention Statin Trials

**Table 2:**
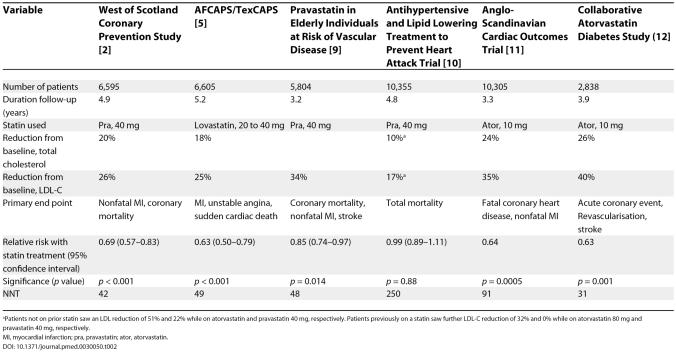
Placebo-Controlled Primary Prevention Statin Trials

**Table 3:**
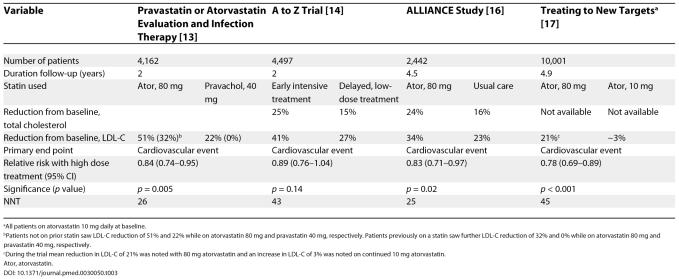
Statin Trials Comparing High-Dose with Low-Dose Regimen

But statins are expensive, and clinicians and policymakers must objectively review the literature so that statin therapy can be appropriately initiated and be cost effective. In developing countries, where a changing lifestyle is increasing the incidence of cardiovascular disease, the need to be cost effective is even more pressing. This paper attempts to derive a fair and evidence-based answer to four practical questions that are especially relevant in less wealthy societies: (1) In whom and when should statin therapy be initiated? (2) What lipid level should physicians aim for? (3) Do different ethnic groups respond differently to statins? and (4) Are statins cost effective?

## In Whom and When Should Statins Be Initiated?

Statins should no longer be seen as treatment for hyperlipidemia, but should be viewed as treatment to reduce and prevent clinical cardiovascular events. Thus, those requiring statins are those at high risk of cardiovascular events, regardless of the baseline lipid levels. In assessing the cardiovascular risk of the presenting patient, lipid levels form only one of the many clinical parameters to be taken into consideration [ 19–21].

A strategy of treatment based on risk will ensure that patients likely to suffer from cardiovascular outcomes will be treated regardless of their initial lipid level, and will avoid unnecessary treatment of the low-risk patient with hyperlipidemia who may not benefit from therapy. The use of lipid level to decide the initiation of treatment must be replaced by the question, “At what cardiovascular risk should statins be started?” Similarly, the individual patient's risk of possible adverse consequences of treatment (hepatitis, myositis, mood changes) should also dictate how cautious the physician should be in initiating and increasing statin usage.

## What Target Lipid Level Should You Aim For?

None of the clinical trials discussed above were designed to answer the question of what lipid level doctors should aim for when prescribing statins to lower a patient's cardiovascular risk. In contrast, other studies, such as the UK Prospective Diabetes Study Group study and the Hypertension Optimal Treatment study, were specifically designed to determine target levels for reduction of risk factors (such as blood pressure and blood glucose) [ 22–24].

Based on a post-hoc review of the major statin trials, the Adult Treatment Panel III of the US National Cholesterol Education Program recently concluded: “In high-risk persons, the recommended low-density lipoprotein cholesterol (LDL-C) goal is < 100 mg/dl, but when risk is very high, an LDL-C goal of < 70 mg/dl is a therapeutic option” [ 25]. This recent advice to seek very low lipid levels of below 70 mg/dl (1.8 mmol/l) for those at especially high risk is thus an extrapolation of the studies, and of epidemiological data, rather than an evidence-based conclusion derived from the trials [ 25, 26].

The larger the LDL-C reduction, the larger the reduction in vascular disease risk, with a reduction of 1 mmol/l of LDL-C over five years reducing major vascular events by 23% [ 18]. Accordingly, a higher dose of statin will lead to a greater reduction in cardiovascular events. However, there is an increased incidence of adverse effects with higher doses of statins [ 13–17]. Thus, the higher-dose statin regime should be reserved for patients at especially high risk of cardiovascular events. The higher the presenting lipid level, the more likely it is that a higher dose of statin can be used.

In any patient, reaching an LDL-C level of 70 mg/dl (1.8 mmol/l) indicates the level at which the statin dosage should not be further increased. The onset of clinical or biochemical adverse effects, or of financial strain upon patients who are having to purchase drugs out of pocket, would similarly suggest that the upper limit of statin dose has been reached. Such an individualized approach to statin therapy reinforces the need for the physician to manage the whole patient clinically, rather than to be excessively distracted by any arbitrarily defined laboratory lipid levels.

## Are There Ethnic Differences in Response to Statins?

The landmark statin trials were conducted in the Western world, and ethnic minorities were greatly underrepresented [ 27, 28]. However, the available data suggest that the correlation between elevated cholesterol and coronary disease holds true for all ethnic groups, including Asians and eastern Europeans [ 29–32]. As people in developing societies attain a more affluent lifestyle and change their dietary habits accordingly, the incidence of dyslipidemia and coronary disease both rise significantly [ 33, 34]. Having said this, the Framingham risk table may not accurately estimate coronary risk amongst Asian patients, and may need to be modified to remain relevant for individuals in developing societies [ 35–37]. Trial evidence must be adapted to suit local populations.

There are ethnic differences in response to drug treatment. African-Americans, for example, are less affected by agents targeting the renin-angiotensin system than by other drugs for treating heart failure [ 38, 39]. A Japanese study of 51,321 patients found that just 5 mg daily of simvastatin reduced total cholesterol by about 20%, and LDL-C by about 25%; these effects persisted for the six years of the trial [ 40]. The US Food and Drug Administration notes that serum levels of rosuvastatin amongst Asians is double that of Caucasians, and has advised that rosuvastatin doses should be halved in Asian patients [ 41].

There is a possibility that smaller and lighter Asians may only require low-dose statin therapy, an idea that would be most welcome in the poorer parts of the world. There are reports that low-dose, alternate-day and even weekly statin therapy can produce efficacious and adequate reduction of lipid levels [ 42–45]. Yet studies conducted with cerivastatin and simvastatin suggest that pharmacokinetic differences between different ethnic groups do not require clinical dosage modification [ 46, 47]. Ethnic differences in treatment response is an area of research that governmental bodies must look into, given the unwillingness of commercial companies to further pursue this route of enquiry.

## Is Statin Therapy Cost-Effective?

Statin therapy of the appropriately high-risk patient has been shown to be cost-effective [ 48–51]. Prevention of cardiovascular disease is less expensive than the treatment of its clinical consequences. However, while curative medicine benefits the symptomatic and ill patient, preventive medicine treats those at risk, who may or may not develop the disease in future. Thus, practical realities of the local community must be remembered when considering preventive statin therapy.

Developing countries are less affluent than the industrialized world, and funds are needed to combat infectious diseases, to provide for maternal and child health care, and to develop good and clean infrastructure for water and food supplies. The nominal per capita gross domestic product of Malaysia is 12% that of the United States (pp. 388 and 607 of [ 52]). In Malaysia, treatment for a year with atorvastatin (Lipitor) 80 mg daily costs RM3,139, which is one sixth of the annual Malaysian gross domestic product per capita of RM18,734. Thus, only a small minority can afford this treatment, while the public health system would be rapidly bankrupted if it were to provide high-dose statin for all patients who might benefit from it.

Daily treatment for a year with Lipitor (patented atorvastatin) 10 mg daily costs RM1,606; the same treatment with Zocor (patented simvastatin) 20 mg daily costs RM1,338; treatment with Lescol (patented fluvastatin) 80 mg daily costs RM1,160; treatment with generic simvastatin 20 mg daily costs RM252; and treatment with generic lovastatin daily 40 mg costs RM183. Since the trials clearly show the beneficial effects of simvastatin and lovastatin (which are available off patent), it is difficult to advocate using patented statins in the developing world [1,5–7]. An alternative strategy would be to purchase a high-dose formulation of the expensive patented statin and break the tablet for daily or alternate-day consumption. Breaking Lipitor 80 mg into quarters and taking it on alternate days, producing an effective dose of atorvastatin 10 mg daily, costs RM392 annually. The health care budget even in the developed world is limited, and funds spent extravagantly mean that some other service would have to be shortchanged.

## Discussion

In objectively reviewing the evidence on the statins, it is important not to miss the forest for the trees. While it is true that some trials of statins for reducing cardiovascular disease have had negative results (e.g., the Antihypertensive and Lipid Lowering Treatment to Prevent Heart Attack Trial [ 10], the A to Z trial [ 14], and the trial by the German Diabetes and Dialysis Investigators [ 53]) nevertheless a meta-analysis involving 90,056 patients did find that statin use was associated with a reduction in cardiovascular end points [ 18].

In the Antihypertensive and Lipid Lowering Treatment to Prevent Heart Attack Trial, in which patients aged 55 years or older, with an LDL-C of 120 to 189 mg/dl (or 100 to 129 mg/dl if they had known coronary heart disease) and triglycerides lower than 350 mg/dl, were randomized to pravastatin or usual care, 32% of usual-care participants with and 29% without coronary heart disease started taking lipid-lowering drugs. The fact that so many usual-care participants took lipid-lowering drugs may have reduced the apparent benefit of the statin taken by the treatment group [ 10]. And although the reduction in primary end point in the A to Z Trial [ 14] (which compared immediate versus delayed treatment with a statin after an acute coronary syndrome) did not reach statistical significance, the number needed to treat to prevent one primary end point (NNT), was 43. This was similar to the NNT of 45 in the Treating to New Targets Trial (17), which produced a highly significant reduction in primary end point ( Table 3). A negative study of statin therapy in 1,255 patients with diabetes on haemo-dialysis [ 53] does not reduce the impact of other, larger trials, involving a total of more than 100,000 patients [ 18, 53, 54]. The point that these three trials emphasizes is that statin treatment should not be blindly started for all patients with hyperlipidemia but requires a thoughtful assessment of the individual patient.

There must now be a shift in the approach to dyslipidemias. From concentrating solely on lipid levels, the emphasis is now on risk stratification, since treatment will be most effective in patients at high risk of clinical cardiovascular disease. The benefit of statin treatment will thus be greater in secondary prevention, where patients have preexisting atheromatous disease and will be more prone to future adverse events. No decision can be made on the need for statin therapy based solely on lipid levels; all therapeutic decisions must be based on the risk the patient has for future cardiovascular events.

The recent studies suggest that a more aggressive statin treatment strategy will lead to greater reductions in cardiovascular events. However, the incidence of side effects from statin treatment, as well as health care costs, increases with the increased dose used. The most potent of the statins, rosuvastatin, is also the one whose adverse effects have caused alarm—some commentators have even called for its withdrawal [ 55–57]. Given the need for continuous treatment, the financial burden of patients on high-dose statins, especially in developing countries, may result in reduced adherence to treatment.

Although patients with established cardiovascular disease who have initial low cholesterol levels have been shown to benefit from treatment, the question of which target level of lipids to aim for remains unanswered. The evidence is clear that in conventional doses studied in the trials, daily simvastatin 10 to 80 mg, pravastatin 20 to 40 mg, atorvastatin 10 to 80 mg, lovastatin 20 to 40 mg, and fluvastatin 80 mg will reduce future cardiovascular outcomes. Yet hyperlipidemia is not the only risk factor predisposing to cardiovascular disease, and lowering cholesterol cannot be the sole approach to battle this health problem. Treatment of other risk factors, such as diabetes and hypertension, as well as lifestyle changes that include increasing physical activity, cessation of smoking, and dietary modification, all have an important role to play in the prevention of cardiovascular disease [ 58]. No medicine is free of adverse effects, and the risk-benefit ratio of treatment in each patient must be carefully considered before initiating statin therapy.

## Conclusion

When deciding whether or not to initiate statin therapy, there should be a shift in emphasis away from the idea of a “normal lipid profile”. Instead, the emphasis should be on the individual patient and the risk factors for cardiovascular disease. The actual threshold for therapy varies between guidelines but the principle remains the same [19–21,35–37]. The higher the risk, assessed from prior atheromatous disease, diabetes, blood pressure, smoking status, age, and sex (besides the lipid levels), the greater the need to treat and to treat aggressively. Indeed, a case can be made for all patients with a prior atheromatous disease to be on a statin, regardless of initial cholesterol level. Patients at low risk of cardiovascular events should not be treated merely because of an abnormal lipid profile, especially since statin treatment is not free from adverse effects. Given the financial costs of statins, it is even more important for physicians with limited resources in developing countries to carefully consider the appropriateness of statin therapy for every patient managed.

## Abbreviations

LDL-C: low-density lipoprotein cholesterol
NNT: number needed to treat to prevent one primary end point
